# The Treatment of Typhoid Fever

**Published:** 1899-12-02

**Authors:** 


### THE TREATMENT OF TYPHOID FEVER.

An interesting article appears in the New York *Medical Record* on "Ten Years' Experience with Typhoid Fever at the Roosevelt Hospital," from the pen of Dr. W. H. Thomson, physician to the hospital. Passing by the many points raised as to the etiology, symptoms, and complications of the disease, we come to the treatment, which involves some peculiarities which are worthy of attention, especially what is said concerning the use of purgatives. On admission, if at any time before the end of the second week, a mercurial purge of gr. 5 of calomel, and gr. 35 of compound jalap powder is administered, and this purgative is repeated every third night till the close of the second week. The presence of diarrhœa is held to be no contra-indication to its administration, for it seems to check it altogether, and to change the character of the evacuations in the majority of cases. It is stated that this treatment appears to directly lower the temperature as its first effect, and to moisten the tongue. In many cases this line of treatment is continued well into the third week, the administration of the purgative only being stopped when there is reason to fear the supervention of intestinal hæmorrhage. The exclusive diet until the fourth week is equal parts of milk and lime water. The use of pepsine is strongly recommended. In typhoid fever the stomach is reduced to the weakness of early infancy in digestive power, so that the milk should always be given diluted, and from the beginning to the end of the fever 10 gr. of pepsine and 10 gr. of bismuth subcarbonate may be given every three hours. Bismuth is looked upon as one of our most serviceable intestinal antiseptics. As soon as the tip of the tongue shows the first sign of dryness 10 to 20 minims of spiritus terebinthinæ are given every four hours in emulsion. In addition to these measures the cold bath is used when the temperature reaches 103 deg. F., and in some cases these baths are charged with the artificial Nauheim salts and carbonic acid. Rectal irrigation with saline solution is employed when there are signs of nephritis with oliguria. The whole number of typhoid fever cases treated by Dr. Thomson at the hospital from September, 1889, to September, 1899, was 368, and the whole number of deaths 25, or 6·8 per cent. Of those who died, however, 14, or 56 per cent., died within a week after admission, many of them being in a hopeless state, if not actually moribund, on their reception.

